# Identification and Analysis of Three Hub Prognostic Genes Related to Osteosarcoma Metastasis

**DOI:** 10.1155/2021/6646459

**Published:** 2021-01-30

**Authors:** Jianye Tan, Haofeng Liang, Bingsheng Yang, Shuang Zhu, Guofeng Wu, Lutao Li, Zhengwei Liu, Lin Li, Weizhong Qi, Sijing Li, Lijun Lin

**Affiliations:** ^1^Department of Orthopedics, Zhujiang Hospital, Southern Medical University, Guangzhou, Guangdong, China; ^2^Department of Orthopedics, Huizhou Municipal Central Hospital, Huizhou, Guangdong, China

## Abstract

Osteosarcoma (OS) often occurs in children and often undergoes metastasis, resulting in lower survival rates. Information on the complexity and pathogenic mechanism of OS is limited, and thus, the development of treatments involving alternative molecular and genetic targets is hampered. We categorized transcriptome data into metastasis and nonmetastasis groups, and 400 differential RNAs (230 messenger RNAs (mRNAs) and 170 long noncoding RNAs (lncRNAs)) were obtained by the edgeR package. Prognostic genes were identified by performing univariate Cox regression analysis and the Kaplan–Meier (KM) survival analysis. We then examined the correlation between the expression level of prognostic lncRNAs and mRNAs. Furthermore, microRNAs (miRNAs) corresponding to the coexpression of lncRNA-mRNA was predicted, which was used to construct a competitive endogenous RNA (ceRNA) regulatory network. Finally, multivariate Cox proportional risk regression analysis was used to identify hub prognostic genes. Three hub prognostic genes (ABCG8, LOXL4, and PDE1B) were identified as potential prognostic biomarkers and therapeutic targets for OS. Furthermore, transcriptions factors (TFs) (DBP, ESX1, FOS, FOXI1, MEF2C, NFE2, and OTX2) and lncRNAs (RP11-357H14.16, RP11-284N8.3, and RP11-629G13.1) that were able to affect the expression levels of genes before and after transcription were found to regulate the prognostic hub genes. In addition, we identified drugs related to the prognostic hub genes, which may have potential clinical applications. Immunohistochemistry (IHC) and quantitative real-time polymerase chain reaction (qRT-PCR) confirmed that the expression levels of ABCG8, LOXL4, and PDE1B coincided with the results of bioinformatics analysis. Moreover, the relationship between the hub prognostic gene expression and patient prognosis was also validated. Our study elucidated the roles of three novel prognostic biomarkers in the pathogenesis of OS as well as presenting a potential clinical treatment for OS.

## 1. Introduction

Osteosarcoma (OS) is characterized by a high recurrence rate and early lung metastasis. It is more prevalent in children and adolescents and is the leading cause of poor survival rates [1]. The disease lesions manifest themselves in the metaphysis of the limb bones [2]. Despite significant advances in adjuvant chemotherapy [3], the high migration and invasion ability of OS antagonizes efforts to reduce its mortality rate [4]. The high mortality rate is associated with the lack of research on the mechanism related to metastatic OS. Understanding the mechanisms of OS metastasis development will not only define important diagnostic or prognostic biomarkers but also inform clinical management of OS.

Recent studies have shown that the competitive endogenous RNA (ceRNA) network, as a theory explaining gene transcription, is closely related to the pathophysiology of many diseases [5–7]. So far, due to its potential biomarker or therapeutic target, the ceRNA network has attracted enormous research interest for clinical application [8, 9]. Research on the ceRNA network and disease metastasis is of particular interest [10–12]. Among the ceRNA, the interplay between long noncoding RNA (lncRNA), messenger RNA (mRNA), and microRNA (miRNA) has been widely investigated. Using molecular sponge activity, lncRNAs attract miRNAs, which then block the interaction between miRNAs and mRNA [13]. This cascade of events thus affects mRNA expression and influences various human disease processes, including OS metastasis [13–15].

Transcription factors (TFs) regulate the transcription process by binding to specific DNA sequences [14–16]. Therefore, TFs affect variability in mRNA expression levels, which ultimately affect various biological processes, including cancer development [17–19]. Regulation by TFs determines the fate of many cells [20]. It has been demonstrated that metastatic OS is also prone to regulation by TFs [21–23].

**Figure 1:**
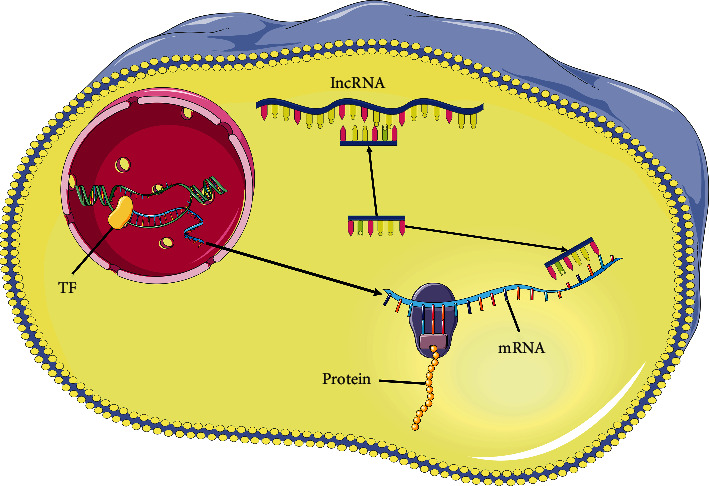
Regulatory patterns of three hub prognostic genes before and after transcription.

We hypothesize that TF, as an RNA-binding protein of mRNA, regulates gene transcription, and lncRNA, as a sponge, interacts with miRNA to influence the translation of mRNA into protein, which in turn leads to tumorigenesis (Figure 1).

In this study, based on the theory that lncRNAs can be used as miRNA sponges to regulate gene expression, a ceRNA network was constructed to find related molecules that affect the development of OS. Moreover, we show that TFs regulate the expression of specific genes and signaling pathways, which define the fate of many cells. These new molecular dynamics may explain the mechanism of OS metastasis. Additionally, we provide targeted therapy agents that are helpful for the treatment of OS patients.

## 2. Materials and Methods

### 2.1. Raw Data

RNA-seq expression profiles (HTSeq-counts) and clinical manifestations of OS were downloaded from The Cancer Genome Atlas (TCGA) database (https://portal.gdc.cancer.gov/repository). The project is TARGET-OS. A total of 88 patients with OS were included, including 66 nonmetastatic patients and 22 metastatic patients.

### 2.2. LncRNA/mRNA Reannotation

To obtain the mRNA and lncRNA expression profiles, the gene transfer format- (gtf-) annotated GRCh38.p12 in the Ensembl database was used to reannotate the downloaded RNA-Seq expression data. Genes with “protein-coding” annotation information were kept as mRNAs. The genes with “3'-overlapping ncRNA,” “ambiguous ORF,” “ncRNA host,” “noncoding,” “processed transcript,” “retained intron,” “sense overlapping,” “sense intronic,” “bidirectional promoter lncrna,” “lincRNA,” or “antisense” were selected as lncRNAs. Clinical phenotypes that corresponded to the gene expression profile samples were also screened.

### 2.3. Differential Analysis

The raw data were normalized by calcNormactors function in the R package, whereas the differences between metastasis and nonmetastasis tumor samples in the two expression profiles were analyzed using the edgeR package [24].

### 2.4. Survival Analysis

The univariate Cox proportional hazards regression analysis and the Kaplan–Meier (KM) survival analysis were used to determine the prognostic genes. Using the multivariate Cox analysis, seven nodes in the ceRNA network were identified that were used to develop a prognosis model. In addition, an algorithm: risk score = *β* gene1∗expr (gene 1) + *β* gene2∗expr (gene 2) + ... + *β* gene∗expr (gene n) was used to calculate the risk score, where *β* is a coefficient for estimating prognosis by Cox analysis, while expr corresponds to the expression value of the gene. The above model was used to obtain a risk score for 88 patients with OS, and the median risk score for all patients was divided into a high-risk group and a low-risk group. The risk survival curve and the five-year survival rate were calculated using the survival R package. The receiver operating characteristic (ROC) [25] curve analysis was used to assess the accuracy of the model for predicting the prognosis and the diagnostic value of gene expression. Data on the risk curves and survival states were processed using the survival package in R.

### 2.5. Coexpression Analysis

The corr.test function in the R package was used to calculate the Pearson correlation coefficient between the prognostic mRNA and the prognostic lncRNA, and the gene pairs were selected (|Pearson correlation coefficient| >0.4 and *P* value <0.001). Similarly, the correlation between the mRNA and TFs was also assessed.

### 2.6. miRNA Regulatory Network

MiRcode [26] (http://www.mircode.org/) was used to predict the intersection between the lncRNAs and miRNAs. StarBase [27] (http://starbase.sysu.edu.cn/), microRNA (http://www.microrna.org/microrna/home.do), and miRDB (http://mirdb.org/) were used to decode the relationship between mRNAs and miRNAs.

### 2.7. Construction of the ceRNA Network

LncRNA-mRNA expression pairs, regulated by the same miRNA, were selected and then integrated with miRNA to construct a ceRNA network. Cytoscape software (version 3.7.2) was used for network construction.

### 2.8. Prediction of TFs

The 1,000 bp upstream sequence of each gene was downloaded from UCSC (http://genome.ucsc.edu/cgi-bin/hgTables). TFBSTooLs and JASPAR2016 packages were used to predict TFs with a gene binding score greater than 0.8.

### 2.9. Function and Pathway Enrichment Analysis

Based on the expression level and median expression values of candidate genes, the OS expression matrix was divided into low and high expression groups. To determine the potential functions of candidate genes, the annotated gene sets c5.all.v7.1.symbols.gm and c2.cp.kegg.v7.1.symbol.gmt were selected as reference. The first five gene sets and pathways as screened by Gene Ontology-gene set enrichment analysis and Kyoto Encyclopedia of Genes and Genomes-gene set enrichment analysis (GSEA) were assessed.

### 2.10. Correlation Analysis of Drug Sensitivity

The CellMiner database (https://discover.nci.nih.gov/cellminer/), a database of 60 cancer cells based on the massive data of drugs and gene targets, was used to identify drugs that are sensitive to cancer cells. The Food and Drug Administration (FDA) and the drug sensitivity data with experimental verification were used to screen *ABCG*8-, LOXL4-, and *PDE*1*B*-related drugs (|Pearson correlation coefficient |>0.4, *P* < 0.01).

### 2.11. OS Patient Data and Tissue Specimens

Data on 31 patients with OS (19 males and 12 females, 16 metastatic and 15 nonmetastatic, median age: 2 years) were collected from the Zhujiang Hospital of Southern Medical University (Guangzhou, Guangdong, China). Informed consent was obtained from all patients before surgery. All experimental procedures were conducted following the Code of Ethics of the World Medical Association (Declaration of Helsinki).

### 2.12. QRT-PCR

Total RNA was extracted from fresh frozen tissues using RNAiso plus reagent (Accurate Biotechnology (Hunan) Co., Ltd., China), and 500 ng of total RNA was reverse transcribed into cDNA. The cDNA was diluted five times with enzyme-free water. One-step qRT-PCR was performed in a 10 *μ*L reaction system. The primers were as follows: *GAPDH* forward: 5'-GGAGCGAGATCCCTCCAAAAT-3', *GAPDH* reverse: 5'-GGCTGTTGTCATACTTCTCATGG-3'; *ABCG*8 forward: 5'-AGCCTCCTTGCTAGATGTGAT-3', *ABCG*8 reverse: 5'- GTCTCTCGCACAGTCAAGTTG-3'; *PDE*1*B* forward: 5'- CTGCGCTACATGGTGAAGCA-3', *PDE*1*B* reverse: 5'- CAAGATTTGCCGTGTCTCATCTA-3'; and LOXL4 forward: 5'- CTGGGCACCACTAAGCTCC-3', LOXL4 reverse: 5'- CTCCTGGATAGCAAAGTTGTCAT-3'. The reaction was performed using the following thermocycling conditions: initial denaturation at 95°C for 30; followed by 40 cycles of 95°C for 30 s and 60°C for 30 s. The relative level of gene expression was calculated using the ^2−ΔΔ^Cq method. Meanwhile, the patients were divided into two groups according to gene expression; those whose expression level was higher than the median were grouped into the high expression group; otherwise, they were classified into the low expression group.

### 2.13. Immunohistochemical (IHC) Staining

Paraffin sections were dewaxed with xylene and rehydrated with ethanol. Tissue sections were incubated in 0.01 mol/L sodium citrate buffer (pH 6.0) for 10 min and 3% hydrogen peroxide for 30 min at room temperature to block endogenous peroxidase. The slides were washed in phosphate-buffered saline (PBS), sealed with 5% bovine serum albumin for 30 min, and incubated with primary antibody overnight in a humidified room at 4°C. Finally, after three washes with PBS, the secondary antibody conjugated with horseradish peroxidase (dilution: 1 : 50; Cat. No. A0208; Beyotime, China) was incubated at room temperature for 60 min, and HRPDAB Kit (Tiangen, China) was used according to the manufacturer's agreement. The main antibodies were ABCG8 (dilution: 1 : 200; Cat. No. DF6673; affinity, China), LOXL4 (dilution: 1 : 200; Cat. No. ab88186; Abcam, China), and PDE1B (dilution: 1 : 200; Cat. No. DF9299; affinity, China). The images were taken with an orthophoto microscope (magnification, 200×). The degree of immunostaining of the specific protein was assessed and labeled by two independent observers. Immunohistochemical (IHC) staining was determined by two parameters, namely, staining intensity and number of cells stained.

### 2.14. Statistical Analysis

All statistical analyses were performed using SPSS statistical software (version 20.0; IBM Corp.). Student's *t*-test was used to evaluate the statistical significance of the difference in the means between the two groups. Survival analysis of different genes was performed using the KM method with the log-rank test. Differences with *P* < 0.05 were considered statistically significant.

## 3. Results

### 3.1. Differential Gene Expression Analysis

**Figure 2:**
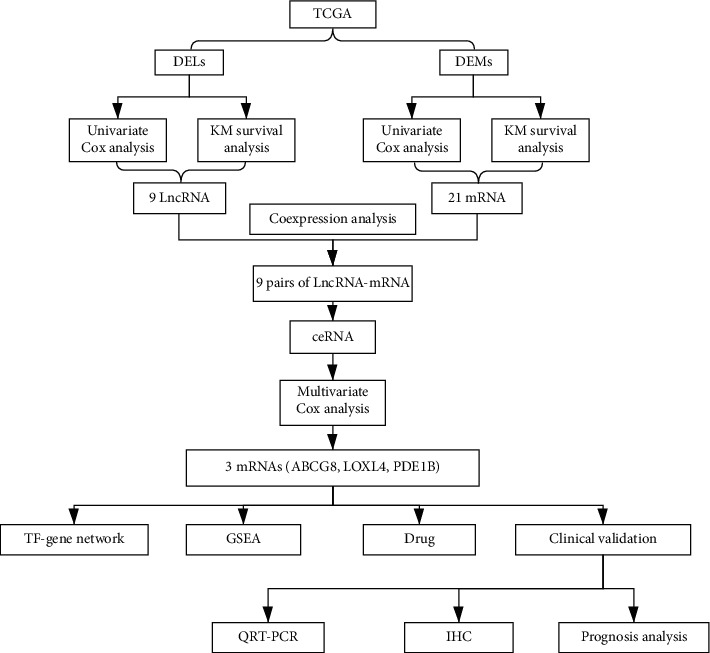
Flow chart of the process of analysis.

**Figure 3:**
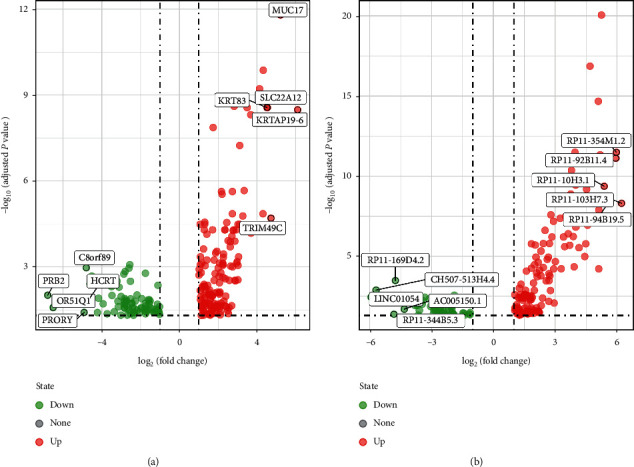
Volcano map of differentially expressed genes. (a, b) Volcano map differential expression mRNAs (top 5) and lncRNAs (top 5). Red represents high expression and green represents low expression.

**Figure 4:**
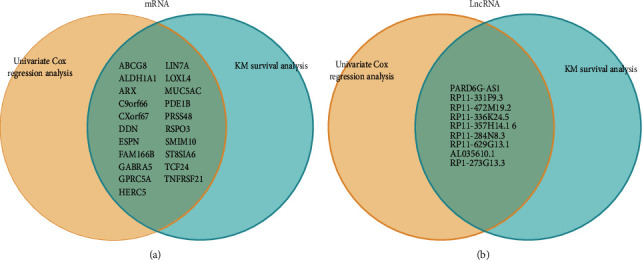
Venn diagram of prognostic genes. (a, b) Univariate Cox regression analysis (the set on the left), KM survival analysis (the set on the right), and prognostic genes studied (the intersecting set).

We studied the high-throughput sequencing data of 88 patients with OS obtained from TCGA and summarized the characteristics of the patients (Table 1). The primary tumor locations of the patients were grouped to identify clinically significant differential gene expression. The flow chart describing this study is shown in Figure 2. The screening thresholds for mRNA and lncRNA were adjusted *P* value <0.05 and |log fold change (FC)|>1. A total of 230 (150 upregulated and 80 downregulated) differentially expressed mRNAs (DEMs) and 170 (123 upregulated and 47 downregulated) differentially expressed lncRNAs (DELs) were identified. The top five genes (in ascending order according to the LogFC) were selected from the DEMs and DELs to draw the volcano map (Figure 3). The univariate Cox regression analysis indicated a correlation between the expression of 49 DEMs and 30 DELs (*P* < 0.05) and OS. Furthermore, using the KM survival curve analysis, we show that the expression of 27 DEMs and 19 DELs (*P* < 0.05) is correlated to OS. Overall, we obtained 30 prognostic genes (21 mRNAs, 9 lncRNAs) (Figure 4).

### 3.2. Analysis of mRNA and lncRNA Coexpression

**Figure 5:**
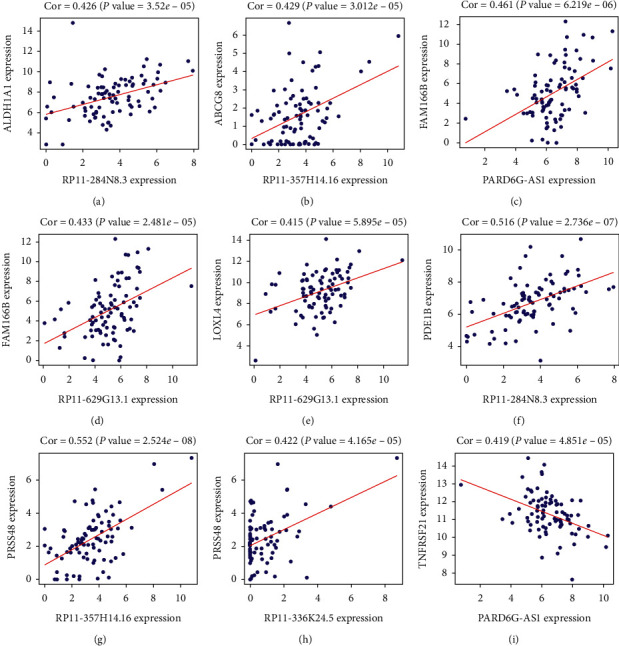
The mRNA-lncRNA coexpression network. The horizontal axis is lncRNA and the vertical axis is mRNA. The correlation and *P* value of lncRNA-mRNA are shown above each graph.

The expression between lncRNAs and mRNAs was determined by Pearson correlation, using the expression profiles of the 9 prognostic lncRNAs and 21 prognostic mRNAs. Our analysis found nine lncRNA-mRNA coexpression pairs (Figure 5). We believe that there is a regulatory relationship between the coexpression of lncRNAs and mRNAs, and mRNAs are the target of coexpression of lncRNAs.

### 3.3. Construction of the ceRNA Network

**Figure 6:**
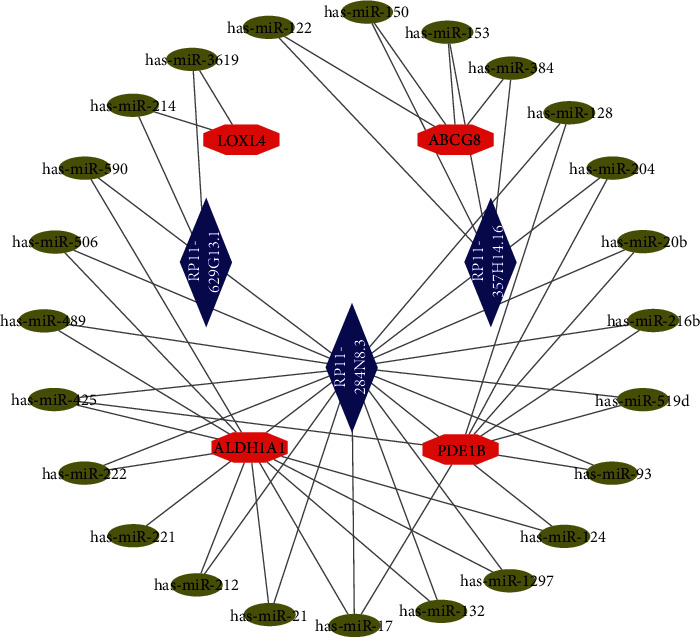
Construction of the ceRNA network. The red node represents mRNAs, the blue node represents lncRNAs, and the yellow node represents miRNAs.

Based on the coexpression relationship between mRNA and lncRNA, lncRNA (RP11-357H14.16, RP11-284N8.3, RP11-629G13.1, RP11-336K24.5) and their combined miRNAs were obtained using the miRcode database. For mRNA with lncRNA-mRNA coexpression relationship, an online database was used to yield miRNAs related to mRNAs (ABCG8, ALDH1A1, LOXL4, PDE1B, FAM166B). Based on the consistent combination of miRNAs with lncRNAs and mRNAs, the ceRNA network was constructed using 4 mRNAs (ABCG8, ALDH1A1, LOXL4, PDE1B), 24 miRNAs, and 3 lncRNAs (RP11-357H14.16, RP11-284N8.3, RP11-629G13.1) (Figure 6). We found that lncRNAs regulate mRNAs through combined miRNAs.

### 3.4. Prognostic Ability of the ceRNA Network

**Figure 7:**
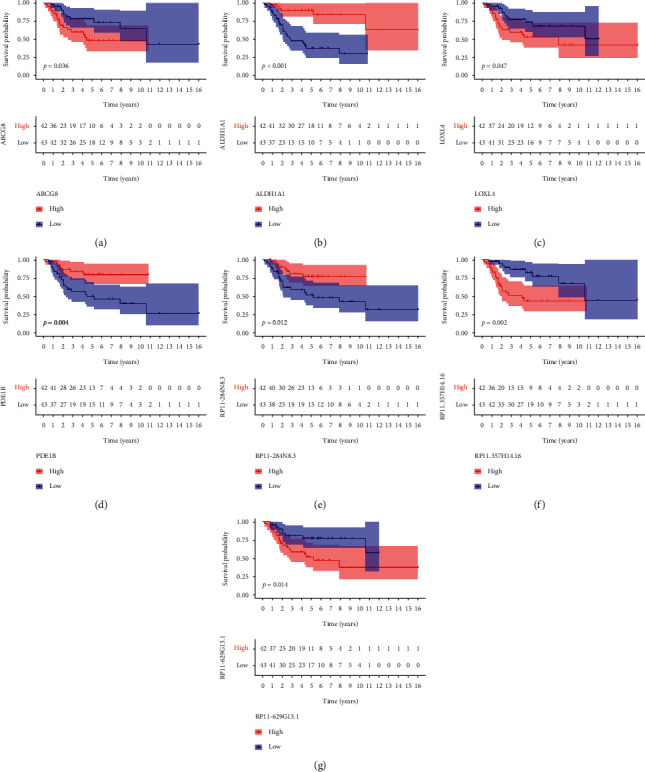
The Kaplan–Meier survival analysis of genes in the ceRNA network.

**Figure 8:**
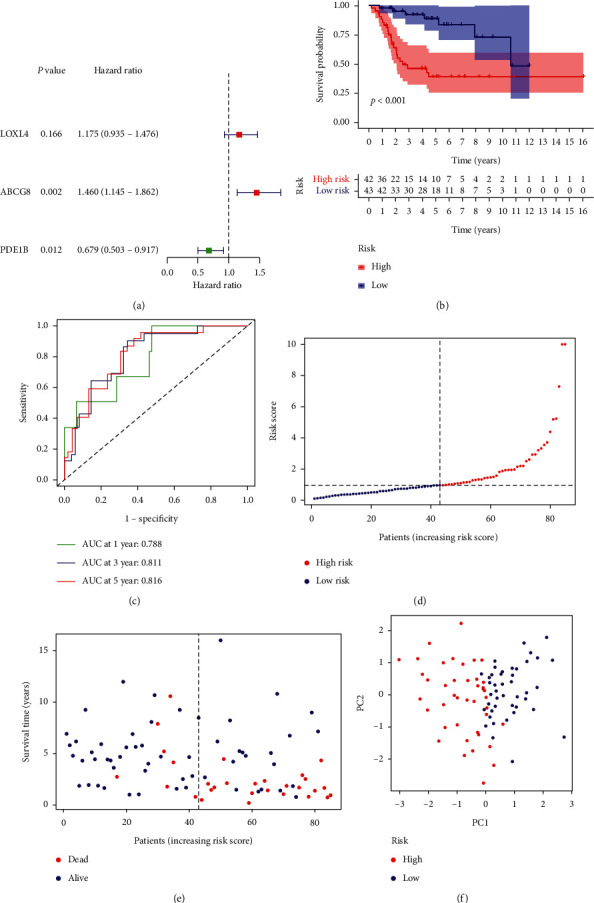
Multivariate Cox regression analysis. (a) Forest graph of multivariate Cox regression analysis. (b) Risk survival curve for the prognostic model. (c) AUC of time-dependent receiver operating characteristic curves verified the prognostic performance of the risk score in the model cohort. (d) Distribution of risk scores of patients. (e) Scatter plot of survival status of patients from different risk groups. (f) Principal component analysis (PCA) diagram of the model cohort.

The KM survival analysis of genes in the ceRNA network is shown in Figure 7. Next, the optimal model for predicting prognosis was determined by the multivariate Cox proportional risk regression analysis, which introduced nodes in the ceRNA network. We found that the results of the multivariate Cox regression model were all mRNAs. We identified ABCG8, LOXL4, and PDE1B as risk evaluation genes in the overall survival model and were considered hub prognostic genes (Figure 8(a)). The mRNA model risk score for overall survival was (0.3787 × ABCG8) + (0.1611 × LOXL4) + (−0.3870 × PDE1B). Our analysis showed that the 5-year overall survival rates were 39.3% or 83.4% for the high- or low-risk groups, respectively (Figure 8(b)). In the ROC curve analysis, the area under the curve (AUC) values for the five-year survival rate based on the gene model was 0.816 (Figure 8(c)). The data robustly demonstrate that our model is a powerful prognostic indicator for OS metastasis. A series of risk curve analyses showed that, as the patient's risk value increased, the survival rate significantly decreased (Figures 8(d) and 8(e)). The principal component analysis showed that patients in different risk groups were distributed in two directions by the constructed model cohort (Figure 8(f)).

### 3.5. Regulation of TFs of Hub Prognostic Genes

**Figure 9:**
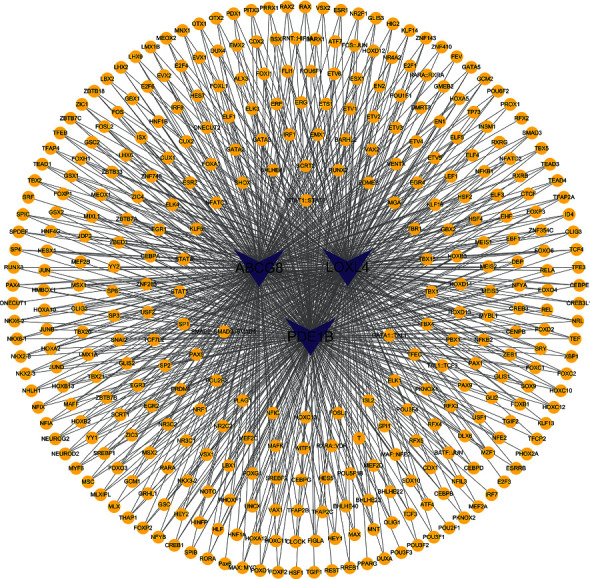
Construction of the TF-gene network. Blue nodes represent genes, and yellow nodes indicate TFs.

**Figure 10:**
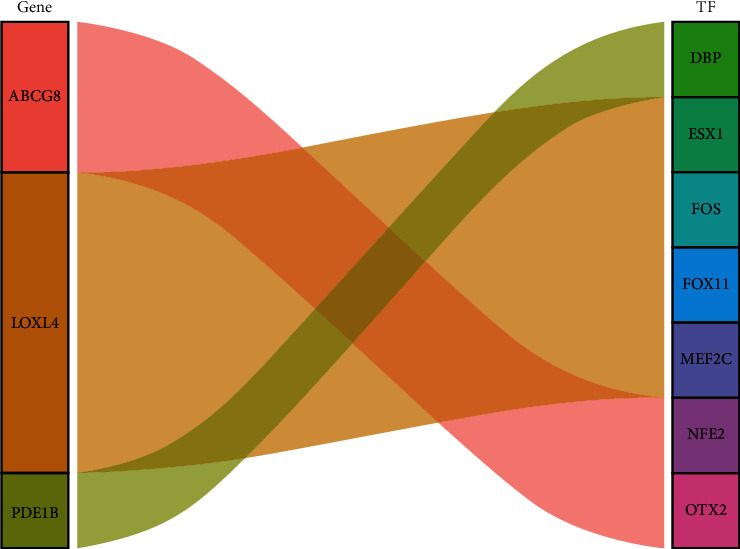
Sankey diagram of TF-gene coexpression.

Prediction of hub prognostic mRNA-binding TFs obtained 361TFs (Figure 9). Further coexpression analysis shows that NFE2 and OTX2 play regulatory roles following ABCG8 transcription (Figure 10). Coexpression of LOXL4 and TFs (MEF2C, FOXI1, FOS, and ESX1) indirectly proves an intersection between their regulatory mechanisms. There is also a strong correlation between PDE1B and DBP. We believe that these TFs coexpressed with genes not only combine with genes upstream to regulate gene expression but also may play a very important role.

### 3.6. Analysis of Single Gene GSEA Enrichment of Hub Prognostic Genes

**Figure 11:**
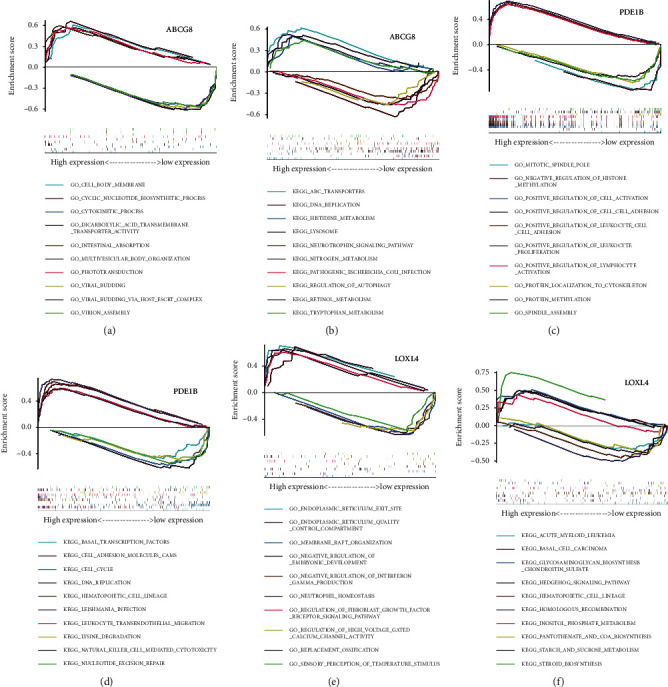
Gene set enrichment analysis.

As stated in the results of the analysis, we observed that gene expression levels extensively influence some pathways to inhibit or promote tumorigenesis and development, thereby revealing the role of hub prognostic genes in OS. Our GSEA of three genes (ABCG8, LOXL4, and PDE1B) revealed that ABCG8 may be related to cellular energy, metabolism, and autophagy, while LOXL4 may be involved in ossification, immunity, and metabolism. PDE1B may be involved in immunity, cell adhesion, methylation, cell cycle, cell metabolism, and TF regulation (Figure 11).

### 3.7. Correlation Analysis of Drug Sensitivity

**Figure 12:**
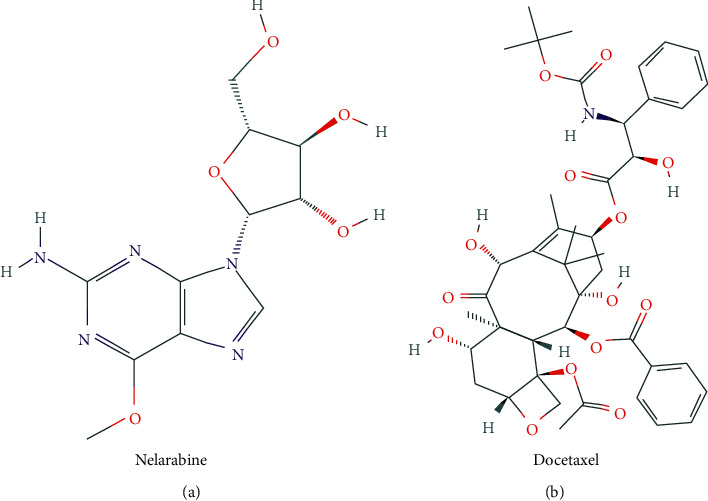
Drug analysis in relation to gene expression.

To further elucidate gene expression and drug sensitivity, which are beneficial to clinical treatment, we obtained drugs that are related to the expression of hub prognostic genes based on previous screening criteria (Supplemental Figure S1). Meanwhile, we searched for the drug most closely related to gene expression and found that PDE1B expression showed a robust positive correlation with nelarabine. LOXL4 has a strong negative correlation with docetaxel, which suggests that the expression of LOXL4 may be related to the unsatisfactory chemotherapy effect in OS patients (Figure 12).

### 3.8. qRT-PCR Verification

**Figure 13:**
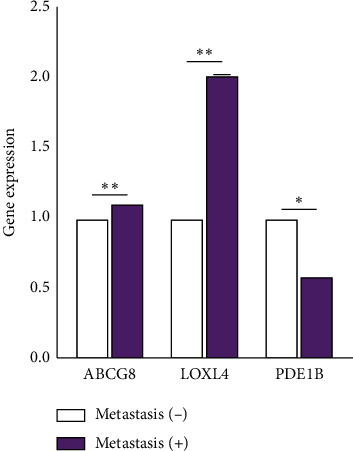
Quantitative real-time polymerase chain reaction results. ^*∗*^*P* < 0.05, ^*∗∗*^*P* < 0.01, ^*∗∗∗*^*P* < 0.001.

To ensure the reliability of our results, we performed qRT-PCR analysis. The results showed that the expression levels of ABCG8 and LOXL4 significantly increased, while those of PDE1B significantly decreased (Figure 13).

### 3.9. IHC verification

**Figure 14:**
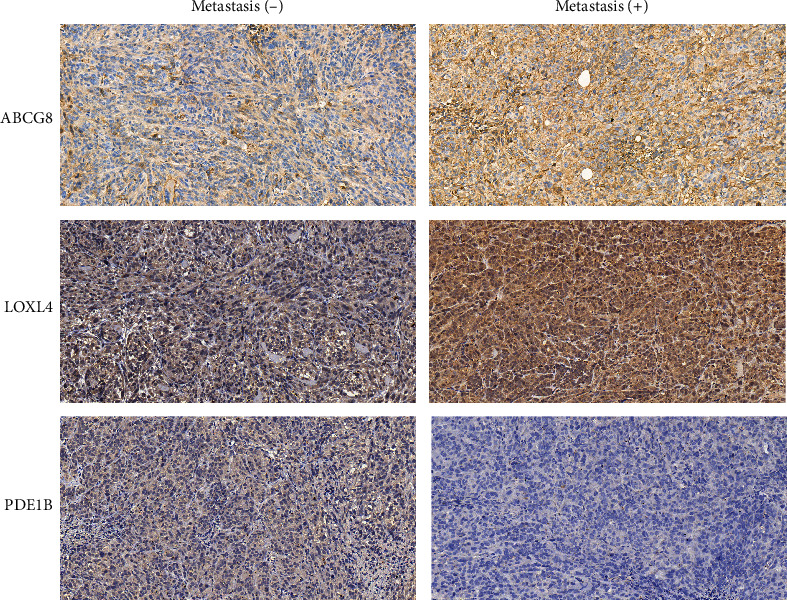
Immunohistochemical staining results.

The protein expression of genes in the prognosis model was evaluated by IHC to provide independent validation of our findings. Compared with metastatic tumor tissues, the expression of ABCG8 and LOXL4 was higher, whereas that of PDE1B was lower, which was concordant to our findings using the TCGA database (Figure 14).

### 3.10. Validation of Clinical Prognosis

**Figure 15:**
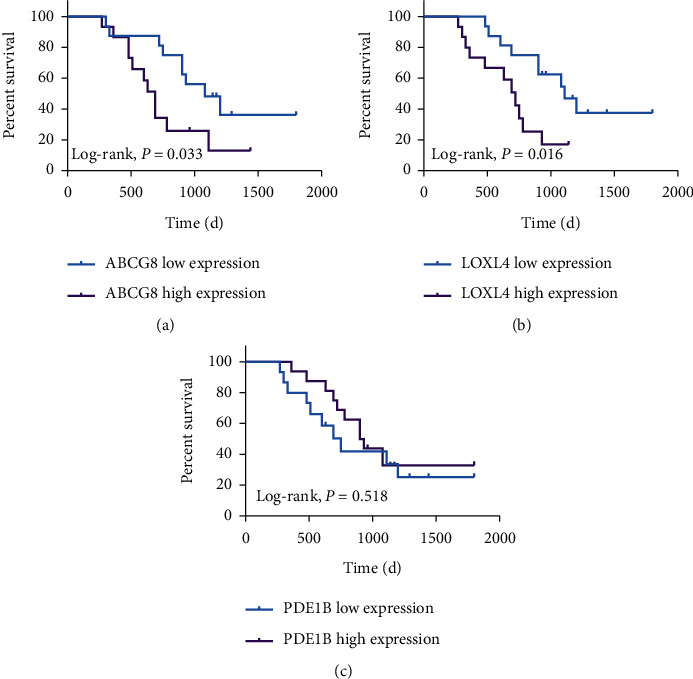
The Kaplan–Meier survival curve of ABCG8, LOXL4, and PDE1B.

Differentially expressed genes were identified using qRT-PCR. To determine whether the expression of hub prognostic genes is correlated to the prognosis of patients with OS, the KM curves of patients with OS with low and high gene expression were analyzed. The results showed that the expression of ABCG8 and LOXL4 was closely related to the survival of patients. This is concordant with our previous results. The survival curves of PDE1B demonstrated a trend toward improving, although the difference was not statistically significant (Figure 15).

## 4. Discussion

OS metastasis is common in children and is associated with high death rates [28]. Therefore, in-depth exploration of the etiology and the pathogenesis of OS, as well as the development of prognostic tools and new treatment regimens, is required. Our study aimed to better understand the mechanisms of metastatic OS in children. Here, we identified 4 mRNAs, 24 miRNAs, and 3 lncRNAs and then constructed a lncRNA-miRNA-mRNA ceRNA network. A better understanding of the intricate interactions among the nodes in the ceRNA network might lead to significant insights into gene regulatory networks, thus influencing cancer treatment. To date, a few studies have evaluated the relationship between ceRNA and OS prognosis. Additionally, rare, yet reliable lncRNAs or mRNA-based biomarkers for OS development are available. To further explore the clinical role of ceRNA, we applied multivariate Cox proportional hazards regression analysis to build a prognostic survival model and determined that three hub prognostic genes (ABCG8, LOXL4, and PDE1B) can reliably predict OS prognosis. Because of metastasis, the prognosis of OS patients will reduce the five-year survival rate to 20%–30% [29]. Our model shows that the five-year survival rate of patients in the high-risk group is as low as 39.3%, while that in the low-risk group is as high as 83.4%. This suggests that these three genes play a very important role in the prognosis of patients, and further exploration of the three genes will be of clinical significance.

We studied the regulatory activities of TFs in tumorigenesis. We analyzed the expression profiles between the TFs and the genes in the transcriptome. The construction of the TF network could further reveal the central prognostic genes, thus showing the cause of metastatic OS. Besides, GSEA analysis revealed that mRNAs may play different roles in OS development. Moreover, the analysis of drug sensitivity strengthened the clinical significance of our research and has a direct effect on the clinical guidance of OS.

The protein encoded by ABCG8 is a member of the adenosine triphosphate- (ATP-) binding cassette transporter superfamily. Previous studies have demonstrated that *ABCG*8 directly or indirectly regulates cholesterol absorption and serum cholesterol level, as well as mediating bone formation. Therefore, the expression of ABCG8 is related to various bone diseases [30, 31]. The upregulation of ABCG8 plays a pivotal role in tumor development and defines disease prognosis [32]. Before transcription, TFs (NFE2 and OTX2) may affect ABCG8 transcription by binding to the upstream sequence of ABCG8. Mechanistically, following transcription, RP11-358H14.16, by acting as a sponge, binds to miRNA, thereby affecting the ABCG8 transcription. It also can be concluded that the effects of ABCG8 on cellular metabolism and autophagy are closely related to OS metastasis.

LOXL4 encodes a member of the lysyl oxidase gene family, which can affect the production of the extracellular matrix. It has been confirmed that the expression of LOXL4 in OS affects cell adhesion; however, data on its extensive biological roles remain limited [33]. LOXL4 regulates tumor metastasis through the FAK/Src pathway to regulate liver cancer angiogenesis and cell-matrix adhesion, resulting in poor prognostic effects [34]. In our study, four TFs (MEF2C, FOXI1, FOS, and ESX1) bind and regulate LOXL4 transcription. Following the transcription, RP11-629G13.1 prevents miRNAs from degrading LOXL4 transcription products by competing with mRNAs in binding miRNAs. Interestingly, the results of GSEA show a similar but more extensive perspective. GSEA analysis shows that the effects of LOXL4 on ossification, immunity, and cellular metabolism are closely related to OS metastasis. Drug sensitivity analysis reveals that the expression of LOXL4 is negatively correlated to survival; similarly, the expression of drugs and LOXL4 is negatively correlated, which suggests that the resistance of chemotherapy drugs to OS may be related to the expression of LOXL4. The main adjuvant chemotherapy regimens for OS include ifosfamide, adriamycin, and cisplatin. However, for patients with refractory OS recurrence and metastasis, the combination of docetaxel and gemcitabine is most commonly used [35]. However, the efficacy of docetaxel in the treatment of OS is still not satisfactory [36]. Our current study may explain this. There is a significant negative correlation between the expression of docetaxel and LOXL4, which suggests that patients with high LOXL4 expression may not be sensitive to docetaxel treatment. Correspondingly, patients with low expression of LOXL4 may have a better therapeutic effect with docetaxel, which provides a new explanation for the adjuvant chemotherapy regimen for clinical patients.

Cell invasiveness and angiogenesis in malignancy appear to have a relationship with PDE1B [37]. For PDE1B, we have a reason to speculate that DBP regulates PDE1B before transcription. Expression of RP11-284N8.3 competes with mRNAs in binding miRNAs, thereby antagonizing the effect of miRNAs on the inhibition of PDE1B expression. Thus, PDE1B can affect the development of OS by regulating immunity, cell adhesion, methylation, cell cycle, cell metabolism, and TFs. It is worth mentioning that PDE1B is the target of FDA-approved drugs, and we also get targeted therapy drugs related to PDE1B gene expression. We further investigated the correlation between PDE1B expression and drug sensitivity and found that PDE1B may be potentially used as a therapeutic target. Nelarabine, a purine analog, has been approved by the FDA for the clinical treatment of lymphoblastic leukemia and lymphoma. It can induce apoptosis and kill tumor cells by destroying DNA synthesis in rapidly dividing cells. Our analysis included the possibility and desirability of nelarabine for the treatment of OS, thus broadening the range of clinical treatment options for OS.

Our research not only found three new mRNAs related to the prognosis of OS for the first time but also identified three novel lncRNAs.

Our findings provide insights into OS prognosis and might be used for personalized follow-up and treatment strategies in the future.

## 5. Conclusions

In summary, we identify and validate a prognostic signature that predicts survival in patients with OS. Our findings have potential applications in the clinical management of OS.

## Figures and Tables

**Table 1:** Clinical characteristics of 88 osteosarcoma patients included in this study.

Characteristic	Females (%)	Males (%)	Total (%)
Age (years)	13.01 ± 3.59	16.73 ± 5.08	15.16 ± 4.86
Race			
Asian	4 (4.55%)	3 (3.4%)	7 (7.95%)
Black	4 (4.55%)	3 (3.4%)	7 (7.95%)
White	19 (21.60%)	33 (37.5%)	52 (59.1%)
Unknown	10 (11.36%)	12 (13.64%)	22 (25%)
Vital status			
Alive	23 (26.14%)	34 (38.64%)	57 (64.77%)
Dead	14 (15.91%)	15 (17.05%)	29 (32.96%)
Unknown	0 (0%)	2 (2.27%)	2 (2.27%)
Metastasis			
Nonmetastatic	25 (28.41%)	41 (46.59%)	66 (75%)
Metastatic	12 (13.64%)	10 (11.36%)	22 (25%)
Primary tumor site			
Leg/foot	33 (37.5%)	47 (53.41%)	80 (90.91%)
Arm/hand	4 (4.55%)	2 (2.27%)	6 (6.82%)
Pelvis	0 (0%)	2 (2.27%)	2 (2.27%)

## Data Availability

The datasets generated for this study can be found in the TCGA database (https://portal.gdc.cancer.gov/repository).

## Supplementary Materials

Supplementary Materials Supplemental Figure S1: drug sensitivity analysis. Supplemental Figure S1: the abscissa is the expression of the gene, and the ordinate is drug sensitivity.

## References

[1] Kager L., Tamamyan G., Bielack S. (2017). Novel insights and therapeutic interventions for pediatric osteosarcoma. *Future Oncology*.

[2] Nie Z., Peng H. (2018). Osteosarcoma in patients below 25 years of age: an observational study of incidence, metastasis, treatment and outcomes. *Oncology Letters*.

[3] Bishop M. W., Janeway K. A., Gorlick R. (2016). Future directions in the treatment of osteosarcoma. *Current Opinion in Pediatrics*.

[4] He X., Gao Z., Xu H., Zhang Z., Fu P. (2017). A meta-analysis of randomized control trials of surgical methods with osteosarcoma outcomes. *Journal of Orthopaedic Surgery and Research*.

[5] Qi X., Zhang D.-H., Wu N., Xiao J.-H., Wang X., Ma W. (2015). ceRNA in cancer: possible functions and clinical implications. *Journal of Medical Genetics*.

[6] Karreth F. A., Pandolfi P. P. (2013). ceRNA cross-talk in cancer: when ce-bling rivalries go awry. *Cancer Discovery*.

[7] Xu J., Li Y., Lu J. (2015). The mRNA related ceRNA-ceRNA landscape and significance across 20 major cancer types. *Nucleic Acids Research*.

[8] Li S., Pei Y., Wang W., Liu F., Zheng K., Zhang X. (2019). Circular RNA 0001785 regulates the pathogenesis of osteosarcoma as a ceRNA by sponging miR-1200 to upregulate HOXB2. *Cell Cycle*.

[9] Zhang S., Ding L., Li X., Fan H. (2019). Identification of biomarkers associated with the recurrence of osteosarcoma using ceRNA regulatory network analysis. *International Journal of Molecular Medicine*.

[10] Rong D., Sun H., Li Z. (2017). An emerging function of circRNA-miRNAs-mRNA axis in human diseases. *Oncotarget*.

[11] Xu R., Rai A., Chen M., Suwakulsiri W., Greening D. W., Simpson R. J. (2018). Extracellular vesicles in cancer - implications for future improvements in cancer care. *Nature Reviews Clinical Oncology*.

[12] Cao M.-x., Jiang Y.-p., Tang Y.-l., Liang X.-h. (2017). The crosstalk between lncRNA and microRNA in cancer metastasis: orchestrating the epithelial-mesenchymal plasticity. *Oncotarget*.

[13] Liu B., Liu J., Liu K. (2019). A prognostic signature of five pseudogenes for predicting lower-grade gliomas. *Biomedicine & Pharmacotherapy*.

[14] Yang Z., Li X., Yang Y., He Z., Qu X., Zhang Y. (2016). Long noncoding RNAs in the progression, metastasis, and prognosis of osteosarcoma. *Cell Death & Disease*.

[15] Wang J. Y., Yang Y., Ma Y. (2020). Potential regulatory role of lncRNA-miRNA-mRNA axis in osteosarcoma. *Biomed Pharmacother*.

[16] Paraskevopoulou M. D., Hatzigeorgiou A. G. (2016). Analyzing MiRNA-LncRNA interactions. *Long Non-coding RNAs*.

[17] Battaglia S., Maguire O., Campbell M. J. (2010). Transcription factor co-repressors in cancer biology: roles and targeting. *International Journal of Cancer*.

[18] Lambert M., Jambon S., Depauw S., David-Cordonnier M.-H. (2018). Targeting transcription factors for cancer treatment. *Molecules*.

[19] Cheng Q., Huang C., Cao H (2019). A novel prognostic signature of transcription factors for the prediction in patients with GBM. *Frontiers in Genetics*.

[20] Takahashi K., Tanabe K., Ohnuki M. (2007). Induction of pluripotent stem cells from adult human fibroblasts by defined factors. *Cell*.

[21] Yang G., Yuan J., Li K. (2013). EMT transcription factors: implication in osteosarcoma. *Medical Oncology*.

[22] Maurizi G., Verma N., Gadi A., Mansukhani A., Basilico C. (2018). Sox2 is required for tumor development and cancer cell proliferation in osteosarcoma. *Oncogene*.

[23] Villanueva F., Araya H., Briceño P. (2019). The cancer‐related transcription factor RUNX2 modulates expression and secretion of the matricellular protein osteopontin in osteosarcoma cells to promote adhesion to endothelial pulmonary cells and lung metastasis. *Journal of Cellular Physiology*.

[24] Robinson M. D., McCarthy D. J., Smyth G. K. (2010). edgeR: a Bioconductor package for differential expression analysis of digital gene expression data. *Bioinformatics*.

[25] Robin X., Turck N., Hainard A (2011). pROC: an open-source package for R and S+ to analyze and compare ROC curves. *BMC Bioinformatics*.

[26] Jeggari A., Marks D. S., Larsson E. (2012). miRcode: a map of putative microRNA target sites in the long non-coding transcriptome. *Bioinformatics*.

[27] Li J.-H., Liu S., Zhou H., Qu L.-H., Yang J.-H. (2014). starBase v2.0: decoding miRNA-ceRNA, miRNA-ncRNA and protein-RNA interaction networks from large-scale CLIP-Seq data. *Nucleic Acids Research*.

[28] Mirabello L., Troisi R. J., Savage S. A. (2009). International osteosarcoma incidence patterns in children and adolescents, middle ages and elderly persons. *International Journal of Cancer*.

[29] Ferguson J. L., Turner S. P. (2018). Bone cancer: diagnosis and treatment principles. *American Family Physician*.

[30] Guan Y., Ackert-Bicknell C. L., Kell B., Troyanskaya O. G., Hibbs M. A. (2010). Functional genomics complements quantitative genetics in identifying disease-gene associations. *PLOS Computational Biology*.

[31] Patel S. B., Graf G. A., Temel R. E. (2018). ABCG5 and ABCG8: more than a defense against xenosterols. *Journal of Lipid Research*.

[32] Hlavata I., Mohelnikova-Duchonova B., Vaclavikova R. (2012). The role of ABC transporters in progression and clinical outcome of colorectal cancer. *Mutagenesis*.

[33] Asuncion L., Fogelgren B., Fong K. S. K., Fong S. F. T., Kim Y., Csiszar K. (2001). A novel human lysyl oxidase-like gene (LOXL4) on chromosome 10q24 has an altered scavenger receptor cysteine rich domain. *Matrix Biology*.

[34] Li R., Wang Y., Zhang X. (2019). Exosome-mediated secretion of LOXL4 promotes hepatocellular carcinoma cell invasion and metastasis. *Molecular Cancer*.

[35] Xu J., Guo W., Xie L. (2018). Combination of gemcitabine and docetaxel: a regimen overestimated in refractory metastatic osteosarcoma?. *BMC Cancer*.

[36] Xiao X., Wang W., Wang Z. (2014). The role of chemotherapy for metastatic, relapsed and refractory osteosarcoma. *Pediatric Drugs*.

[37] Chatterjee G., Rosner A., Han Y. (2002). Acceleration of mouse mammary tumor virus-induced murine mammary tumorigenesis by a p53172H transgene. *The American Journal of Pathology*.

