# Supplementary material for: Identification and Analysis of Three Hub Prognostic Genes Related to Osteosarcoma Metastasis
Source: J Oncol. 2021 Jan 30;2021:6646459. doi: 10.1155/2021/6646459 (PMC7867449; doi:10.1155/2021/6646459)
Supplement: Supplementary Materials — Supplemental Figure S1: drug sensitivity analysis. Supplemental Figure S1: the abscissa is the expression of the gene, and the ordinate is drug sensitivity. [file 6646459.f1.pdf]

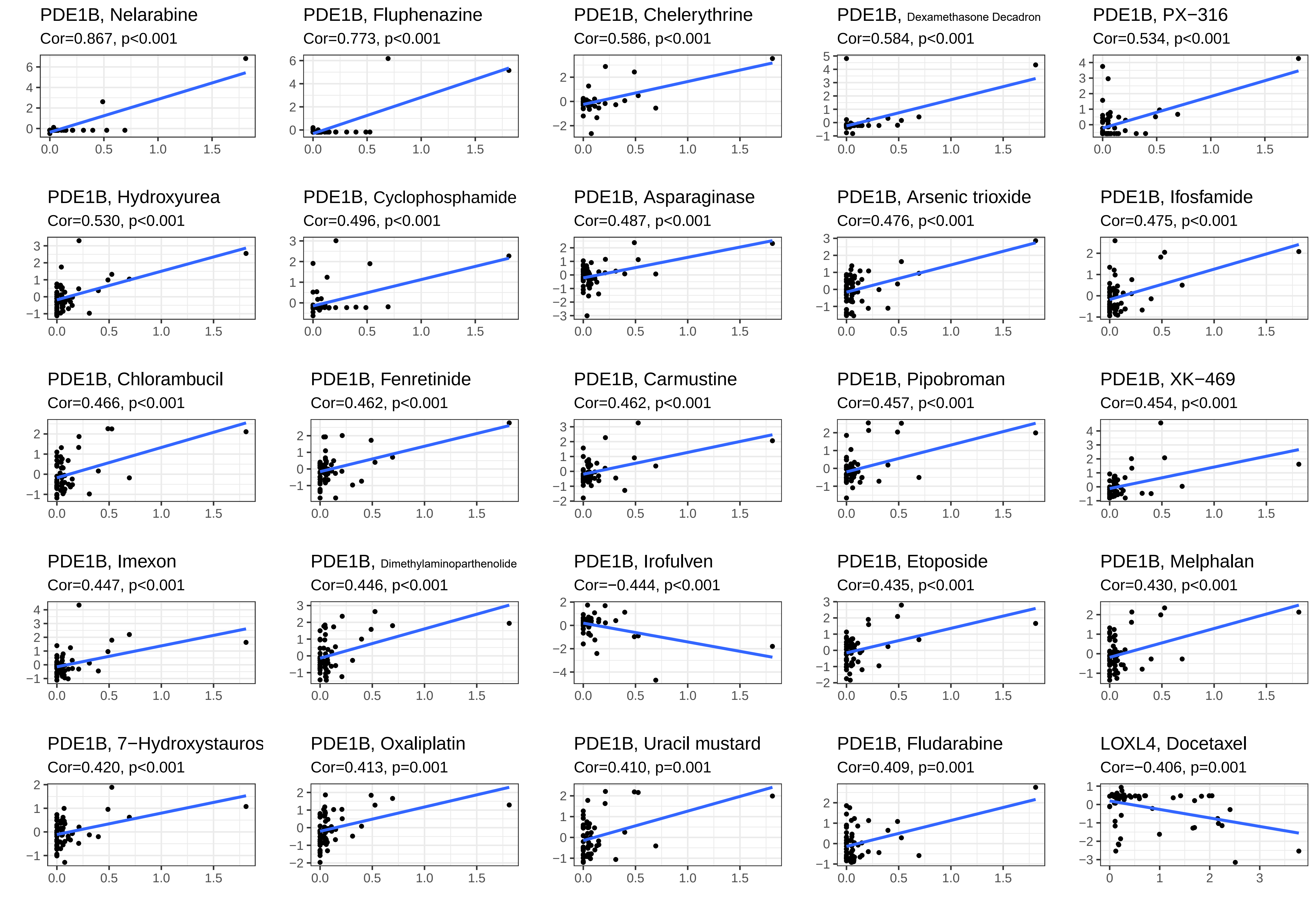
PDE1B, Nelarabine
Cor=0.867, p<0.001
PDE1B, Fluphenazine
Cor=0.773, p<0.001
PDE1B, Chelerythrine
Cor=0.586, p<0.001
PDE1B, Dexamethasone Decadron
Cor=0.584, p<0.001
PDE1B, PX−316
Cor=0.534, p<0.001
PDE1B, Hydroxyurea
Cor=0.530, p<0.001
PDE1B, Cyclophosphamide
Cor=0.496, p<0.001
PDE1B, Asparaginase
Cor=0.487, p<0.001
PDE1B, Arsenic trioxide
Cor=0.476, p<0.001
PDE1B, Ifosfamide
Cor=0.475, p<0.001
PDE1B, Chlorambucil
Cor=0.466, p<0.001
PDE1B, Fenretinide
Cor=0.462, p<0.001
PDE1B, Carmustine
Cor=0.462, p<0.001
PDE1B, Pipobroman
Cor=0.457, p<0.001
PDE1B, XK−469
Cor=0.454, p<0.001
PDE1B, Imexon
Cor=0.447, p<0.001
PDE1B, Dimethylaminoparthenolide
Cor=0.446, p<0.001
PDE1B, Irofulven
Cor=−0.444, p<0.001
PDE1B, Etoposide
Cor=0.435, p<0.001
PDE1B, Melphalan
Cor=0.430, p<0.001
PDE1B, 7−Hydroxystauros
Cor=0.420, p<0.001
PDE1B, Oxaliplatin
Cor=0.413, p=0.001
PDE1B, Uracil mustard
Cor=0.410, p=0.001
PDE1B, Fludarabine
Cor=0.409, p=0.001
LOXL4, Docetaxel
Cor=−0.406, p=0.001
